# Remarks on the Modus Operandi of Calomel

**Published:** 1859-05

**Authors:** Hayden Coe

**Affiliations:** Atlanta, Georgia


### ARTICLE III.

*Remarks on the Modus Operandi of Calomel.*—By HAYDEN COE, M.D., of Atlanta, Georgia.

There is no article in the materia medica which has had a wider range in the treatment of diseases than Calomel. And certainly nothing can be more essential, than that, (an agent which has had and still holds, such high rank in the cure of disease, one which is so extensively used,) its *modus operandi* should be well understood, and its effects upon the various tissues should be thoroughly investigated. Although it has been well known to the profession for more than two centuries, yet, the mode of its operation, and the effects which it has upon the tissues is still unsettled. For it has been placed in the materia medica as a sialagogue, a stimulant, a sedative, a tonic, revulsive, and as a hyposthenics, according to the opinion of the author, or the prevailing theory of the day, which only shows that it, like many other agents, produces different effects, according to the dose administered.

No article of the materia medica has been assailed with so much violence, as has this powerful agent, by the ignorant and the empyric. They have used all the means which they could command to excite and prejudice the people against this remedy, in which they have succeeded, in some limited circles, for a time. But, like all other powerful agents, in the hands of the scientific and skilful, it is a safe and harmless agent, but in the hands of the ignorant charlatan, or the empyric, it is liable to be used to the injury of those to whom they administer it. And, I ask, what other agent, which is capable of producing decided effect upon the tissues of the system, is not liable to be abused in the same way, if they who are ignorant of the nature and operation of the human system, attempt to use it? True, say they, but then there is homœopathy! it is so safe, so mild, so agreeable to the patient, there is no danger in it. I will admit that there is much truth in the above assertion, and of all the systems of humbugery and quackery that have been gotten up, none are so well calculated for the use of the ignorant, and those who have neither the disposition nor the ability to acquire a knowledge of anything, as Homœopathy. It has advantages which are possessed by no other system of humbuggery, for the use of any agent, however simple, might produce some injurious effect if repeatedly used by those who are entirely ignorant of the human system and the effect of their agents upon the tissues of which the system is composed. But to have a system which requires you to administer nothing at all, or at least, only the one-billionth of a drop, or less, so that it will have no effect at all upon the system, is a system of practice that is the best calculated for the ignorant; one in which they are likely to do less harm, I think, than any other that ever has been or ever can be gotten up. And so long as the people remain totally ignorant of the nature and operation of their own bodies, they will remain incapable of appreciating the value of any agent which may be used for the cure of disease. But once more, and then I will return to my subject. Is it more profitable to the patient to die for the want of the use of an agent that would subdue the disease, than to die from the use of the agent which was given for the removal of the disease? Do our agents which we use for the removal of disease, produce a more deleterious effect on the system than the disease itself? If so, then let us throw aside our medicine, say it is all a humbug! yea, worse than a humbug, for it is a poison, it is worse than the morbific agent which produced the disease. Then let us practice homœopathy to lull the mind, and trust entirely to nature to do the work. But, we believe that the long experience of ages, and by men of as good talent as the world has ever afforded, has established beyond a doubt, that agents, and powerful agents, too, in the hands of those who have studied their profession thoroughly, those that are well acquainted with the human system, and the nature of their remedies, and the effect that they will produce on the system, are not only not injurious, but are capable of relieving the system of disease, and are invaluable to the human family. But to return to our subject.

Calomel has long been used under the common term of an alterative, a term that might be as appropriately applied to any other remedy as calomel; for any agent that is capable of making an impression upon the system, must alter the action of some organ or tissue. But the term alterative has been confined to such remedies as are capable of effecting a change in the action of some organ or tissue without making any very manifest impression on the system. The effects of calomel upon certain tissues, as the glandular and mucous, are very well known to the profession, for some of the changes which have been effected are obvious, as its action on the glandular system, promoting secretion, and in larger doses producing purgation; it also seems to act as a sedative in an irritable condition of the mucous membrane of the stomach and bowels. But the mode or manner in which it effects these changes seems to have received much less attention from the profession, and to be much less understood. The composition and properties of calomel are so well known and so well understood by the profession, I deem it wholly unnecessary for me to say anything upon this part of the subject, and shall confine the balance of my remarks exclusively to its *modus operandi.* Neither will it be proper for me, in this article, if I were capable, to enter into an argument upon the general *modus operandi* of medicines, whether they act by absorption, or through the medium of the nervous system, or in both these ways. But how calomel, which is insoluble and very heavy, could be absorbed from the stomach, so that three or four grains could reach all the glandular tissues, the whole extent of the mucous membrane, and produce its effects by contact with the tissues, or how it could so effect a change in the whole mass of blood, as it must do, if it acts through the medium of the blood, that is, it must so change the constituents of the blood, that the blood itself is an irritant to these tissues, instead of a nutrient, or else serves as a vehicle to convey the calomel to these tissues, if the absorption of calomel is necessary to the production of its usual effects. That calomel may be absorbed in small quantities, where it has been administered for a length of time, I shall not attempt to deny; but that three or four grains of calomel, and it an insoluble substance, is capable of being absorbed, and of changing the whole mass of blood, or of being brought in contact with all the tissues it affects, is a mystery which I have never been able to solve satisfactorily to my mind; and I think it will require a powerful stretch of the imagination of any man to imagine, without attempting to reconcile, the effects produced by this small amount of calomel, with the theory of absorption. Each tissue has its own peculiar irritability, a property of which every tissue in the body is endowed, peculiar to itself, and has its own peculiar stimulus, which acts on that irritability, producing normal action in that tissue, ergo, the blood is the stimulus to the heart, the urine to the bladder, the feces to the bowels, &c. But suppose we change the natural stimulus for an unnatural one, and it acts on the irritability of the tissue, will it not change the action from normal to abnormal, and is this not just what takes place, for pathology is only altered physiology, or abnormal action brought about by some unnatural stimulus, which we call a morbific agent. Again, therapeutic action is only altered action, or a change of action in the tissue, which has been produced by the application of a therapeutic agent, which is only another stimulus to the irritability of the tissue effecting another change in its action.

It is a law well established in Physiology, that the irritability of a tissue is acted on by a stimulus or irritant, in two ways, viz: 1st. By the direct application of the stimulus, or irritant to the tissue itself, let the mode by which it is brought in contact with the tissue be what it may. 2d. Through the medium of the nervous system, that is, a stimulus or irritant applied to the nerves which supply the tissue, will act on the irratability of the tissue, producing action in the tissue, as if the irritant had been applied directly to the tissue itself. Therefore, an irritant applied to a portion of the ganglionic nerves, makes an impression which is conveyed to a ganglion, which acts as a nervous center, and from these nervous or ganglionic centers, the impression is diffused, until it reaches the tissues to which they (the nerves) are distributed, affecting the irritability of the tissue precisely as if the irritant had been applied directly to the tissue itself. Hence, calomel is a stimulus applied to the ganglionic nerves, which are distributed to the mucous membrane of the stomach, which impression is conveyed to the semilunar ganglion, and from thence to the glandular and mucous tissues, which are abundantly supplied with ganglionic nerves. But as we have already said, each tissue has its own peculiar irritability, and one agent may affect the irritability of one tissue, and another agent the irritability of another tissue, precisely the effect we witness in our patients daily. It seems to be the property of calomel to act on the irritability of the glandular and mucous tissues, as a stimulus. It seems to have such a decided effect on these tissues, some of them at least, that if long continued, actual inflammation will follow as the result of this excited irritation in these tissues, produced by this agent, and the irritability in these tissues is so changed, and is in such an excitable state, that cold, or even the minutest dose of mercury is sufficient to excite the irritability of these tissues to such an extent as to be followed by inflammation. An agent might act upon the irritability of the tissues as a stimulus or depressant, but calomel is evidently a stimulant, its effect upon the nerves themselves is not so well known, and probably its tendency is to increase innervation. Hence, we see why it is so essential in cases where we wish to bring the system under its specific influence, that we reduce the system by blood letting or otherwise, unless the system has been previously reduced by disease, for if we do not we cannot control it. Its action will be excessive, and as a consequence do serious injury to our patient. Calomel is a valuable agent in all diseases where there is a sluggish action in the glandular system, and in all our adynamic diseases, where there is a want of sensibility, or rather irritability; yet in our typhoid fevers, in most cases at least, although it is an adynamic disease, and there is a want of glandular action; yet, from the simple fact that the mucous membrane of the intestinal canal is in an irritable or inflamed condition, calomel is not admissable.

Cholera, and in cholera morbus, and in cholera infantum, and other adynamic diseases of a kindred character, there is not this irritable or inflamed condition of the mucous membrane. There is in these cases a great want of inervation, as is very manifest, and as a consequence, a stagnation in the function of the liver, the circulation in the abdominal viscera is obstructed. Now, an agent that would increase innervation, and excite the irritability of the glandular tissue, would be what we want. Hence, calomel would be a valuable agent. The increase of the discharge from the bowels in these diseases is not from irritation of the mucous membrane, but is an exudation, owing to the congestion of the vessels, produced by an abstraction in the portal circulation. Whenever calomel is administered in large doses, the impression made on the nerves is such that it extends to the irritability of the muscles of the intestinal canal, exciting mobility, and is so effectually removed from the system in this way, that its action on the glandular tissue is not excessive when administered in large doses, unless it is retained in the system by opium. But it always acts on the glandular tissues, whether administered in small or large doses, as is manifest even when it acts as a cathartic.

I have extended this article to much greater length than I expected, although I feel that much more might be said on this subject, and I hope what I have said may induce some one more able to investigate this subject thoroughly.

